# Adapting the EAT-Lancet diet for West Africa: protein quality and micronutrient inadequacies improved through nutrient dense foods

**DOI:** 10.3389/fnut.2025.1673484

**Published:** 2026-01-14

**Authors:** Hannah Sanders, Didier Y. Alia, Jonathan Lara-Arevalo, Ty Beal, Adam Drewnowski

**Affiliations:** 1 Food Systems Nutrition and Health Program, School of Public Health, University of Washington, Seattle, WA, United States; 2 Daniel J. Evans School of Public Policy and Governance, University of Washington, Seattle, WA, United States; 3 Department of Nutrition, Gillings School of Global Public Health, University of North Carolina at Chapel Hill, Chapel Hill, NC, United States; 4 Global Alliance for Improved Nutrition, Washington, DC, United States; 5 Center for Public Health Nutrition, University of Washington, Seattle, WA, United States

**Keywords:** EAT-Lancet planetary health diet, West Africa Food Composition Tables (WAFCT), Nutrient Rich Food (NRF) index, nutrient-rich foods, protein quality, micronutrient adequacy

## Abstract

**Background:**

The EAT-Lancet planetary health diet was designed as a universal nutritionally adequate diet with minimal environmental impact. We aim to assess and propose revisions to increase its nutrient adequacy in the context of West Africa based on the local food supply.

**Methods:**

We created a model EAT-Lancet diet using nutrient composition data from the FAO's Food Composition Tables for Western Africa (WAFCT). Median energy and nutrient profiles of EAT-Lancet diet food groups were calculated using WAFCT foods (*n* = 596). Protein content was adjusted using the Protein Digestibility Corrected Amino Acid Score (PDCAAS). We multiplied the recommended EAT-Lancet diet intake for each food subgroup by these medians to determine daily nutrient intake. Nutrient adequacy was determined based on alignment with the FAO Codex nutrient reference values for adults. The Nutrient Rich Food (NRF) index, based on priority micronutrients, defined nutrient density. Isocaloric revisions were made to the EAT-Lancet diet to enhance its nutrient adequacy using WAFCT foods.

**Results:**

Total energy of the modeled diet was 2,516 kcal/day. Total protein was 87 g/day while PDCAAS corrected protein was 62 g/day. Micronutrient shortfalls were observed for zinc, calcium, and vitamin A but not for iron, folate, and vitamin B12. Increasing intake of nutrient-rich liver, small dried fish, and pulses, while reducing whole grains and tree nuts, achieved micronutrient adequacy.

**Conclusion:**

When analyzed using foods available in West Africa, the EAT-Lancet diet may provide adequate protein but not vitamin A, zinc, and calcium. Future iterations of the diet should consider including categories for micronutrient dense foods to ensure adequacy.

## 1 Introduction

The EAT-Lancet planetary health diet (EAT-Lancet diet) was designed by the EAT-Lancet Commission (the Commission) to be a nutritionally sufficient diet optimized to prevent diet-related chronic diseases, while minimizing environmental impacts of food production (1). The EAT-Lancet diet contains limited animal-source foods, favoring whole grains, legumes, nuts, vegetables, fruits, and unsaturated oils (1). Despite offering a universally-applicable diet, the Commission recognized the importance of adapting it to local foodways and differences in sex and individual health status. Following its launch, several higher income countries have localized the EAT-Lancet diet to promote plant-forward dietary guidelines (2, 3).

West Africa is currently experiencing a double burden of malnutrition characterized by persistent undernutrition and growing diet-related chronic disease prevalence (4, 5). Despite some progress toward the Sustainable Development Goal of ending all forms of malnutrition by 2030 (6), West Africa has a disproportionately high prevalence of protein inadequacy and shortfalls in dietary iron, zinc, calcium, vitamin A, vitamin B12 and folate (5, 7, 8). [M]Most children under five and women of reproductive age (62% and 80%, respectively) in sub-Saharan Africa are deficient in at least one micronutrient (8). Consequently, there is a high burden of anemia among women of reproductive age in West Africa (5). Any proposed diet needs to take the needs of these populations into account.

Evaluating the EAT-Lancet diet adequacy in the West-African context is essential, as promoting limited animal-source foods for this region and other low- and middle-income countries could be counterproductive to efforts aimed at addressing persistent undernutrition (5, 9). On average, typical diets in West Africa are heavily plant-based with minimal consumption of red meat and dairy (10). Therefore, most dietary protein is plant-sourced. Adapting the EAT-Lancet diet to West Africa also raises several potential concerns. First, the EAT Lancet diet, as currently published, does not provide estimates for protein and does not consider protein quality. Despite its environmental benefits, dietary protein from plants is typically of lower quality compared to animal-source proteins (11). Protein quality shown as the proportion digestible is commonly assessed using the Protein Digestibility Corrected Amino Acid Score (PDCAAS) or the Digestible Indispensable Amino Acid Score (DIAAS) (11, 12). Though the DIAAS is preferred (12), data for individual foods are largely missing. As a result, PDCAAS is more useful in whole diet analysis.

Second, the EAT-Lancet diet may present micronutrient shortfalls, hence adherence to the diet could perpetuate deficiencies already experienced in the region. Using a custom complied global food database, the EAT-Lancet diet has been shown to be inadequate in iron, zinc, calcium and vitamin B12 for all adults and even less adequate in women of reproductive age (13). The study proposed micronutrient adequacy (without fortification or supplementation) could be achieved by increasing the proportion of animal source foods and reducing foods high in phytates.

Building on this concept and responding to the Commission's call for local adaptation of the EAT-Lancet diet, our goals were to: (1) assess the nutritional adequacy of the diet using the FAO/INFOODS Food Composition Table for Western Africa (WAFCT), with adjustments for protein quality, and (2) revise the EAT-Lancet diet using regional foods that are both nutrient-dense and culturally acceptable.

## 2 Methods

### 2.1 The FAO food composition table for Western Africa

The FAO Food Composition Table for Western Africa was selected for a very specific reason. The database lists regional foods that are consumed in a number of countries including Benin, Burkina Faso, The Gambia, Ghana, Guinea, Mali, Niger, Nigeria and Senegal. Food names are available in English and in French. The foods selected for inclusion are representative of the region, are frequently consumed, and are culturally acceptable by definition. The use of regional databases is critical when it comes to evaluating the feasibility of dietary guidelines with a global reach, such as the EAT-Lancet Planetary Health Diet.

To place the EAT-Lancet diet in the context of West Africa, we used the WAFCT which lists 1,028 foods and beverages, along with their energy and nutrient content per 100 grams, edible portion (14). The version of WAFCT downloaded had multiple items with missing or lower quality (i.e., due to doubtful data or different analytical methods) nutrient values. Missing energy and nutrient values of raw foods or their equivalents were obtained from the USDA Food and Nutrient Database for Dietary Studies 2017–2018 nutrient composition database (15). Foods with missing values that could not be matched were excluded (*n* = 116) (16). Foods with lower-quality nutrient values, as defined by the FAO, were retained (*n* = 205).

The WAFCT foods were manually coded into eight groups and 21 subgroups that followed the EAT-Lancet categorization scheme (1). Although this approach may have led to exclusions that risk underrepresenting foods in their most commonly consumed forms, we prioritized following the Commission's guidance in categorizing foods to enhance comparability. When criteria for EAT-Lancet diet subgroups lacked definitions, such as red and orange vegetables, we applied the closest food group criteria from the related FAO Minimum Dietary Diversity for Women (17). When not specified, foods in all forms available in the WAFCT were included, notably including both raw and cooked vegetables, tubers, and starchy vegetables. Following the EAT-Lancet specification that whole grains and legumes be dry, cooked grains and legumes were excluded (*n* = 134). The EAT-Lancet diet proposed 250 g per day of dairy products defined as whole milk or derivative equivalents. To simplify calculations, we included only whole milk (3.5% dairy fat) rather than calculating equivalents in terms of protein, calories or calcium. We excluded processed items in the WAFCT that were specifically excluded by EAT-Lancet, such as refined grains, fortified foods, infant formula, and sugary beverages. Every EAT-Lancet subgroup was represented in the WAFCT except for lard or tallow (set at 5 g/day). The final analytical sample contained 596 foods with complete data for energy, protein and micronutrient content (Table 1).

**Table 1:** WAFCT food items with groupings mapped into EAT-Lancet, Beal et al., and Model-WAFCT categories.

**EAT-Lancet food group**	**EAT-Lancet food subgroup**	**Beal et al. (13) category**	**Model-WAFCT subcategory**	**Count**
Whole grains (rice, wheat, corn, and other)	Whole grains (rice, wheat, corn, and other)	Whole grains	Millet	16
			Maize	12
			Rice	4
			Wheat	4
			Sorghum	3
			Fonio	2
			Oat	1
			Teff	1
		Refined grains	Refined grains (excluded)^*^	56
**Total**				**43**
Tubers or starchy vegetables (potatoes and cassava)	Tubers or starchy vegetables (potatoes and cassava)	Tubers or starchy vegetables	Cassava	11
			Plantain	20
			Potato	5
			Sweet Potato	4
			Yam	31
			Other starchy vegetables	9
**Total**				**80**
Legumes	Dry beans, lentils, and peas	Dry beans, lentils, and peas	Dry beans, lentils, and peas	25
	Soy foods	Soy foods	Soya bean	6
**Total**				**31**
Vegetables	Dark green leafy vegetables	Dark green leafy vegetables	Dark green leafy vegetables	64
	Red and orange vegetables	Red and orange vegetables	Red and orange vegetables	19
	Other vegetables	Other vegetables	Other vegetables	35
**Total**				**118**
Fruits	All fruit	All fruit	Red and orange flesh fruits	5
			Other fruits	39
**Total**				**44**
Nuts	Peanuts	Peanuts	Peanuts	12
	Tree nuts	Tree nuts	Tree nuts	8
Seeds	Seeds	10
**Total**				**30**
Beef, lamb, and pork	Beef and lamb	Beef and lamb	Lamb/mutton	4
			Goat	8
			Other meat	14
		Beef	Beef	17
		Organs (liver, spleen, kidney, and heart)	Beef and small ruminant organs (spleen, kidney, and heart)	11
		Beef and small ruminant liver	8
Pork	Pork	Pork	12
**Total**				**74**
Chicken and other poultry	Chicken and other poultry	Chicken and other poultry	Chicken and other poultry	25
		Organs (liver, spleen, kidney, and heart)	Chicken organs (spleen, kidney, and heart)	4
		Chicken liver	4
**Total**				**33**
Eggs	Eggs	Eggs	Eggs	14
**Total**				**14**
Fish	Fish	Fish	Prepared fish	60
		Fresh fish	Fresh fish	20
		Small dried fish	Small dried fish	1
		Canned fish with bones	Canned fish with bones	5
		Crustacean	Crustacean	13
		Bivalves	Bivalves	7
**Total**				**106**
Dairy foods (whole milk or equivalents)	Dairy foods (whole milk or equivalents)	Whole milk or derivative equivalents	Whole milk	1
**Total**				**1**
Unsaturated oils	Unsaturated oils	Unsaturated oils	Unsaturated oils	8
**Total**				**8**
Saturated oils	Palm oil	Palm oil	Palm oil	4
	Dairy fats (in milk)	Dairy fats (in milk)	Dairy fats (included in milk)	3
	Lard or tallow	Lard or tallow	Lard or tallow	0
**Total**				**7**
All sugars	All sugars	All sugars	All sugars	3
**Total**				**3**
**Grand total**				**596**

^*^Refined grains are included in the Beal et al. (13) analysis but excluded in the EAT-Lancet diet.

We followed Beal et al. (13) in identifying nine additional subgroups of high nutrient density foods (Table 1). This scheme further delineated beef and lamb into beef and organ meats, and fish into fresh fish, small dried fish, canned fish with bones, crustaceans, and bivalves. A new category of seeds was added. We disaggregated particular subgroups even further to inventory the WAFCT and better reflect differences in micronutrient content (18). Red and orange flesh fruits were separated from other fruits. Whole grains, tubers and starchy vegetables, meat, and organs were separated by type.

### 2.2 Adding protein quality adjustment using PDCAAS

PDCAAS values for individual food items or food groups were obtained from the literature and were applied to WAFCT individual foods and groups and were applied similarly across raw and cooked forms (Supplementary Table 1). PDCAAS correction values were applied to 578 of 596 items. Unadjusted protein was multiplied by PDCAAS values for a final adjusted protein content. The remaining 18 items include oils and sugars that contain little to no protein; therefore, PDCAAS is not applicable.

### 2.3 Modeling the EAT-Lancet diet

Recommended daily intakes for each EAT-Lancet diet subgroup were the starting point for modeling analyses (1). When ranges were provided, we used the midpoint. Other than dairy foods, EAT-Lancet diet subgroups were composed of multiple items in the WAFCT (Table 1). The analyses used median energy in kilocalories (kcal), protein, and micronutrient values per 100g for each category. Using means would have biased the results toward outliers and weighting means based was not possible without additional data. We followed numerous precedents in calculating energy and nutrient content of the modeled EAT-Lancet diet food subgroups based on total mass consumed per day (19, 20). Beal et al. (13) alternatively calculated nutrient content of each food groups by matching the energy in kcals per day rather than by mass. We applied this method for sensitivity analyses; the results were not meaningfully different, likely because the total energy of the mass-based analysis was nearly identical to that of the EAT-Lancet diet (Supplementary Table 2).

### 2.4 Energy and nutrient adequacy standards

Despite the variability of individual energy needs, the EAT-Lancet diet targets a daily intake of 2,500 kcal, which corresponds to daily energy expenditure of a 70 kg male and 60 kg female with a moderate to active physical activity level (1). Nutrient reference values (NRV) for protein and micronutrients came from the FAO Codex Alimentarius for adults (Supplementary Table 3) (21). The values used for zinc and iron were based on 22% and 10% dietary absorption, respectively, in a cereal-based, low animal protein diet (21). though actual absorption rates vary based on phytates, polyphenols, and other nutrients in the food matrix (22).

### 2.5 The Nutrient Rich Food (NRF) nutrient density score

The Nutrient Rich Food (NRF) index was the main measure of nutrient density (23). The NRF index was applied to the EAT-Lancet subgroups and three additional subgroups, chicken liver, beef and small ruminant liver, and small dried fish, which were selected based on their exceptional scores in other analyses (18). The present version, the NRF index 6.3 (NRF6.3), consisted of a positive NR6 subscore based on six nutrients to encourage, and a negative LIM subscore based on three nutrients to limit. The micronutrients to encourage were iron, zinc, calcium, vitamin A [as retinol activity equivalents (RAE)], vitamin B12, and folate [as dietary folate equivalents (DFE)]. The three nutrients to limit were saturated fat, sodium, and added sugar. Percent daily values (%DV) for each nutrient were calculated per 100 kcal using the NRV and were capped at 100%. The NRF6.3 score was the sum of %DV for the six nutrients to encourage minus the sum of %DV for the three nutrients to limit.

### 2.6 Plan of analysis

The recommended EAT-Lancet diet intake (in g/day) for each food subgroup was multiplied by median values for energy and nutrient content of that subgroup from WAFCT. Nutrient retention factors were included in the database according to FAO methodology (13). Nutrient intakes were compared to the NRVs, and any difference was used to determine nutrient adequacy (positive values) or inadequacy (negative values). A linear programming analysis was executed to determine a revised diet that achieves adequacies for all six micronutrients within the EAT-Lancet's original energy intake. Subsequent isocaloric modifications of the EAT-Lancet diet modeled the effects of adding food subgroups that had the highest NRF6.3 scores. Possible solutions were generated by adding these subgroups until the NRV for all micronutrients was reached. Subgroups with low NRF6.3 scores were reduced to realign the intake with the original calorie goal. All calculations were performed without using optimization software, thus the revised diet presented is one of many possible solutions. Analyses were conducted using Microsoft Excel (Version 2503) and R (Version 4.3.1).

## 3 Results

### 3.1 Modeled energy, protein, and adjusted protein content

Table 2 shows the recommended weights, energy, protein, and adjusted protein for each food group in the EAT-Lancet diet compared to the model based on WAFCT medians. The WAFCT model estimates daily energy at 2,516 kcal/day, which is nearly identical to the EAT-Lancet diet at 2,503 kcal/day. In the WAFCT model, 88% of calories came from plants and 12% came from animal-source foods; the EAT-Lancet was 86% and 14%, respectively.

**Table 2:** Daily weight energy, protein and adjusted protein of the EAT-Lancet diet and modeled diet components based on median food group and category values from the WAFCT.

**EAT-Lancet food group**	**EAT-Lancet food subgroup**	**EAT-Lancet (g/d)**	**EAT-Lancet (kcal/d)**	**Model-WAFCT (kcal/d)**	**Model-WAFCT Protein (g/d)**	**Model-WAFCT PDCAAS protein (g/d)**
Whole grains	Whole grains (rice, wheat, corn)	232	811	812	21.81	9.54
Tubers or starchy vegetables	Tubers or starchy vegetables (potatoes and cassava)	50	39	68	0.90	0.67
Vegetables	Dark green leafy vegetables	100	23	46	4.25	3.11
	Other vegetables	100	25	36	1.40	0.80
	Red and orange vegetables	100	30	56	1.50	1.11
Fruits	Fruits	200	126	141	1.90	1.13
Dairy foods	Dairy foods (whole milk)	250	153	160	8.25	8.25
Protein sources	Beef and lamb	7	15	13	1.81	1.70
	Pork	7	15	25	1.48	1.45
	Chicken and poultry	29	62	44	7.40	6.95
	Eggs	13	19	23	1.66	1.66
	Fish	28	40	33	6.52	6.13
	Dry beans, lentils, peas	50	172	161	10.40	7.18
	Soy foods	25	112	96	8.76	7.97
	Peanuts	25	142	144	5.60	2.63
	Tree nuts	25	149	137	3.55	1.67
Added fats	Palm oil	6.80	60	61	0	0
	Unsaturated oils	40	354	360	0	0
	Dairy fats (in milk)	0	0	0	0	0
	Lard or tallow	5	36	0	0	0
Added sugar	All sugars	31	120	101	0.12	0.12
Plant-source total (%)		985 (74)	2,163 (86)	2,217 (88)	60.19 (69)	35.92 (58)
Animal-source total (%)		339 (26)	340 (14)	299 (12)	27.12 (31)	26.15 (42)
Grand total		1,324	2,503	2,516	87.32 (34.6 g/1,000 kcal)	62.07 (24.5 g/1,000 kcal)

Total protein of the WAFCT model was 87 g/day, of which 60 g (69%) were plant-source and 27 g (31%) were animal-source. The estimated amount of digestible protein dropped to 62 g/day following PDCAAS adjustment. This was equivalent to a drop from 34.6 g/1000 kcal/day in the WAFCT model to 24.6 g/1000 kcal/day in the WAFCT PDCAAS-adjusted model. Whole grains, dry beans, lentils, and peas; and peanuts were the categories that exhibited the largest reduction in protein due to low PDCAAS values. Following the PDCAAS adjustment, 36 g (58%) of digestible protein came from plants and 26 g (42%) came from animal-source foods.

### 3.2 Priority micronutrient shortfalls

Table 3 shows estimated priority micronutrients in the WAFCT model by food subgroup. Vitamin A, zinc, calcium did not meet the NRV. Estimated vitamin A was 61% of the NRV at 488 μg RAE. Dark green leafy vegetables and red and orange vegetables contributed the majority of vitamin A, supplying 210 μg and 127 μg, respectively. Estimated zinc was 13.88μg, 99% of the NRV. Whole grains supplied 5.10μg of zinc. Estimated daily calcium was 864 mg, 86% of the NRV. Dairy foods (milk) and dark green leafy vegetables contributed 298 mg and 249 mg of calcium, respectively.

**Table 3:** Modeled micronutrient content of the EAT-Lancet diet based on median values from the WAFCT.

**EAT-Lancet food group**	**EAT-Lancet food subgroup**	**EAT-Lancet (g/d)**	**Model-WAFCT totals**					
			**Folate, DFE (**μ**g/d)**	**Iron (mg/d)**	**Calcium (mg/d)**	**Vitamin B12** **(**μ**g/d)**	**Zinc (**μ**g/d)**	**Vitamin A, RAE (**μ**g/d)**
Whole grains	Whole grains (rice, wheat, and corn)	232	164.72	15.78	53.36	0	5.10	0
Tubers or starchy vegetables	Tubers, starchy vegetables (potatoes and cassava)	50	8.75	0.48	7.50	0	0.22	2.50
Vegetables	Dark green leafy vegetables	100	66.00	3.70	249.00	0	0.69	210.00
	Other vegetables	100	20.50	1.00	29.00	0	0.27	9.00
	Red and orange vegetables	100	15.00	1.80	33.00	0	0.30	127.00
Fruits	Fruits	200	27.00	1.30	43.00	0	0.30	7.00
Dairy foods	Dairy foods (whole milk)	250	20.00	0.25	297.50	1.48	1.58	105.00
Protein sources	Beef and lamb	7	0.60	0.22	1.26	0.17	0.25	0.60
	Pork	7	0.56	0.15	0.98	0.03	0.14	0.14
	Chicken and other poultry	29	1.45	0.41	3.77	0.11	0.59	6.38
	Eggs	13	8.45	0.31	9.17	0.22	0.20	15.47
	Fish	28	3.36	0.42	16.80	0.95	0.31	3.92
	Dry beans, lentils, and peas	50	165.00	2.30	37.00	0	1.20	0.50
	Soy foods	25	95.00	1.73	51.75	0	1.20	0
	Peanuts	25	27.50	0.90	12.63	0	0.65	0.50
	Tree nuts	25	14.50	1.38	14.63	0	0.82	0
Added fats	Palm oil	6.8	0	0.01	0.07	0	0	0
	Unsaturated oils	40	0	0.03	0	0	0	0
	Dairy fats (included in milk)	0	0	0	0	0	0	0
	Lard or tallow	5	0	0	0	0	0	0
Added sugar	All sugars	31	0.62	0.19	3.41	0	0.06	0
Grand total			**639.01**	**32.34**	**863.81**	**2.95**	**13.88**	**488.01**
Nutrient reference value (19)			400	22	1,000	2.4	14	800
Difference in nutrient content and NRV (% of NRV)			**+239.01 (160)**	**+10.34 (147)**	–**136.19 (86)**	**+0.55** **(123)**	–**0.12 (99)**	–**312.00 (61)**

Folate, iron and vitamin B12 were adequate. Estimated folate was 6391 μg, 160% of the NRV. Over half of the folate came from dry beans, lentils and peas, and whole grains, which each contributed 165 μg. Estimated iron was 32.34 mg, 147% of the NRV for a high phytate diet. Much of the iron came from whole grains (15.78 mg/day). Estimated vitamin B12 was 2.95 μg, 123% of the NRV. Whole milk contributed over half (1.48 μg) of daily requirement.

### 3.3 Identifying the most nutrient rich food subgroups

Nutrient density for the EAT-Lancet diet and select Model-WAFCT subgroups was evaluated using the NRF6.3 (Supplementary Table 4). Those with the highest NRF6.3 scores were chicken liver (391.24), beef and small ruminant liver (307.22), dark green leafy vegetables (203.33), small dried fish (200.80) and fish (140.78). The subcategories with the lowest NRF6.3 scores were palm oil (−37.11), all sugars (−35.08), dairy fats (−23.19), unsaturated oils (−11.23) and tree nuts (8.01).

### 3.4 Revisions to improve nutrient density of the EAT-Lancet diet

Table 4 shows potential isocaloric improvements of the EAT-Lancet diet based on foods in the WAFCT. The revisions included adding minimal amounts of chicken liver (2 g), beef and small ruminant liver (2 g), and small dried fish (7 g), increasing dry beans, lentils, and peas by 50 g, and reducing whole grains by 32 g and tree nuts by 15 g. Following these modifications, PDCAAS-adjusted protein intake increased to 71.6 g/day, with the amount from animal sources rising to 44%. The ratio of animal- to plant-source energy remained the same.

**Table 4:** Energy and protein content of the revised EAT-lancet diet based on WAFCT median values.

**EAT-Lancet food group category**	**EAT-Lancet food subgroup**	**Net change (g) (% change)**	**Revised EAT-lancet (WAFCT) (g/d)**	**Revised model-WAFCT energy (kcal/d)**	**Revised model-WAFCT PDCAAS protein (g/d)**
Whole grains	Whole grains (rice, wheat, and corn)	−32 (−14)	200	700	8.22
Tubers or starchy Vegetables	Tubers or starchy vegetables (potatoes and cassava)	0 (–)	50	68	0.67
Vegetables	Dark green leafy vegetables	0 (–)	100	46	3.11
	Other vegetables	0 (–)	100	36	0.80
	Red and orange vegetables	0 (–)	100	56	1.11
Fruits	Fruits	0 (–)	200	141	1.13
Dairy foods	Dairy foods (whole milk)	0 (–)	250	160	8.25
Protein sources	Beef and lamb	0 (–)	7	14	1.82
	Beef and small ruminant liver	+2 (200)	2	4	0.47
	Pork	0 (–)	7	25	1.45
	Chicken and other poultry	0 (–)	29	44	7.03
	Chicken liver	+2 (200)	2	3	0.44
	Eggs	0 (–)	13	23	1.66
	Fish	0 (–)	28	33	6.13
	Small dried fish	+7 (700)	7	22	3.97
	Dry beans, lentils, and peas	+50 (33)	100	321	14.35
Soy foods	0 (–)	25	96	7.97
Peanuts	0 (–)	25	144	2.63
Tree nuts	−15 (−60)	10	55	0.67
Added fats	Palm oil	0 (–)	6.8	61	0.00
	Unsaturated oils	0 (–)	40	360	0.00
	Dairy fats (included in milk)	0 (–)	0	0	0.00
	Lard or tallow	0 (–)	5		0.00
Added sugar	All sugars	0 (–)	31	101	0.12
Plant-source total (%)			992.80 (76)	2,184 (87)	40.78 (56)
Animal-source total (%)			345.00 (14)	328 (13)	31.24 (44)
Grand total			**1,338.80**	**2,512**	**72.02**

**Figure 1:**
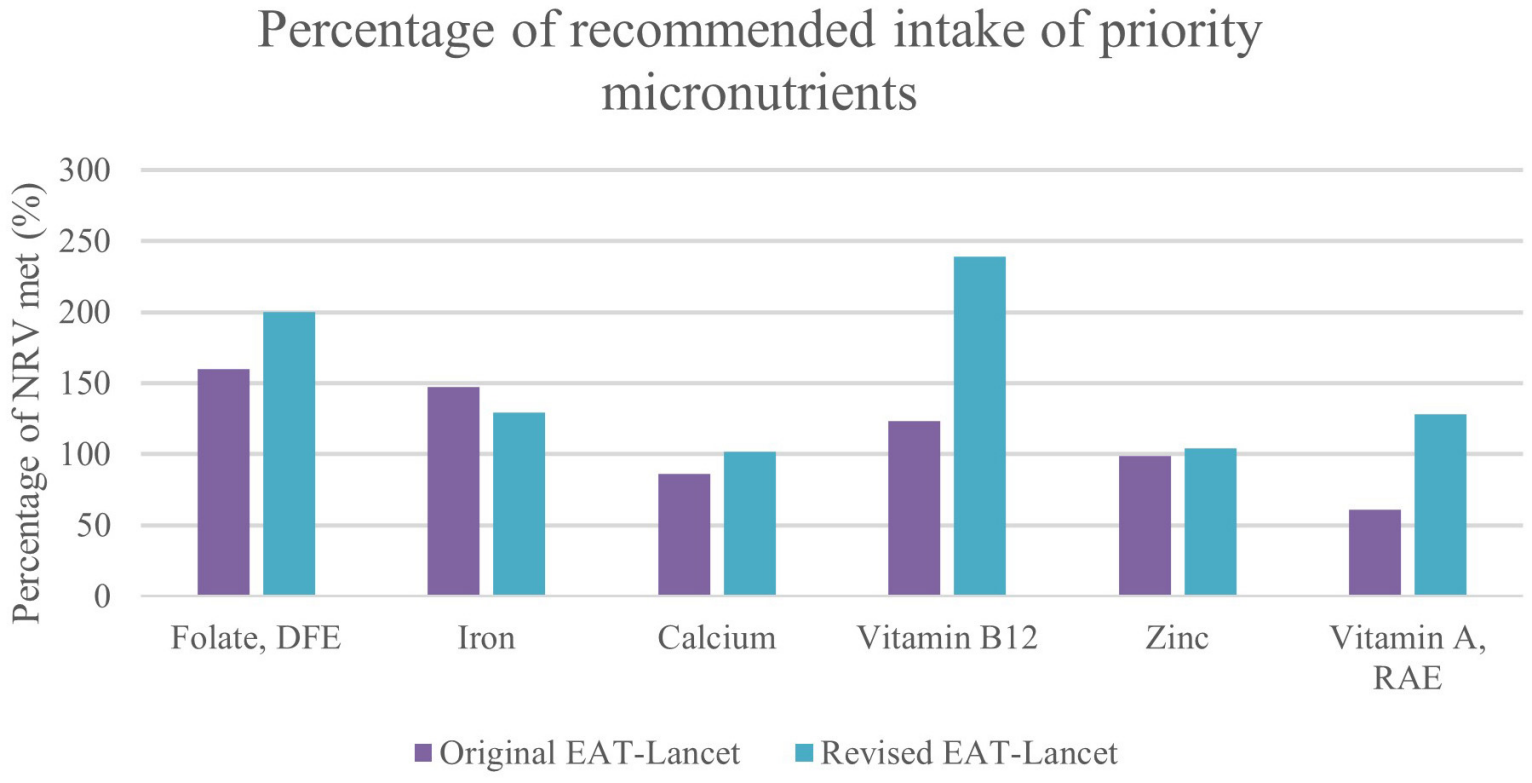
Percentage of priority micronutrient intake in original and revised EAT-Lancet diet based on WAFCT median values.

Table 5 shows that all micronutrients evaluated meet their respective NRV under the revised diet. Increased legume intake contributed to zinc adequacy since legumes had the highest zinc content of any EAT-Lancet food group (4.40 μg/d). Chicken liver and beef and small ruminant liver are extremely dense in vitamin A; therefore, including these categories separately from their respective meat contributed to nutritionally adequate vitamin A (Supplementary Tables 4, 5). Small dried fish are high in calcium, with a median of nearly 2,000 mg per 100 g. Therefore, including this category in the modeled diet led to adequate calcium intake. Though vitamin B12 was sufficient in the original EAT-Lancet diet, intake more than doubled with the 11 g increase in animal-source products. The differences between micronutrient intake in the original and revised EAT-Lancet diet are visualized in Figure 1.

**Table 5:** Micronutrient content of the revised EAT-Lancet diet based on WAFCT median values.

**EAT-Lancet food group**	**EAT-Lancet food subgroup**	**Net change (g; % change)**	**Revised EAT-Lancet intake (g/d)**	**Revised model-WAFCT totals**					
				**Folate, DFE (**μ**g/d)**	**Iron (mg/d)**	**Calcium (mg/d)**	**Vitamin B12 (**μ**g/d)**	**Zinc (**μ**g/d)**	**Vitamin A, RAE (**μ**g/d)**
Whole grains	Whole grains (rice, wheat, and corn)	−32 (−14)	200	142.00	13.60	46.00	0	4.40	0
Tubers or starchy vegetables	Tubers or starchy vegetables	0 (–)	50	8.75	0.48	7.50	0	0.22	2.50
Vegetables	Dark green leafy vegetables	0 (–)	100	66.00	3.70	249.00	0	0.69	210.00
	Other vegetables	0 (–)	100	20.50	1.10	29.00	0	0.27	9.00
	Red and orange vegetables	0 (–)	100	15.00	0.90	33.00	0	0.30	127.00
Fruits	Fruits	0 (–)	200	27.00	1.30	43.00	0	0.30	7.00
Dairy foods	Dairy foods (whole milk)	0 (–)	250	20.00	0.25	297.50	1.48	1.58	105.00
Protein sources	Beef and lamb	0 (–)	7	0.42	0.23	1.09	0.16	0.24	0.49
	Beef and small ruminant liver	+2 (200)	2	4.70	0.25	0.43	2.00	0.08	366.00
	Pork	0 (–)	7	0.56	0.15	0.98	0.03	0.14	0.14
	Chicken and other poultry	0 (–)	29	1.16	0.35	3.77	0.09	0.51	4.06
	Chicken liver	+2 (200)	2	20.00	0.24	0.14	0.45	0.07	162.20
	Eggs	0 (–)	13	8.45	0.31	9.17	0.22	0.20	15.47
	Fish	0 (–)	28	3.36	0.42	16.80	0.95	0.31	3.92
	Small dried fish	+7 (700)	7	3.22	0.52	135.73	0.35	0.54	9.87
	Dry beans, lentils, and peas	+50 (33)	100	330.00	4.60	74.00	0	2.40	1.00
Soy foods	0 (–)	25	95.00	1.73	51.75	0	1.20	0
Peanuts	0 (–)	25	27.50	0.90	12.63	0	0.65	0.50
Tree nuts	−15 (−60)	10	5.80	0.55	5.85	0	0.33	0
Added fats	Palm oil	0 (–)	6.8	0	0.01	0.07	0	0	0
	Unsaturated oils	0 (–)	40	0	0.03	0	0	0	0
	Dairy fats (included in milk)	0 (–)	0	0	0	0	0	0	0
	Lard or tallow	0 (–)	5	0	0	0	0	0	0
Added sugar	All sugars	0 (–)	31	0.62	0.19	3.41	0	0.06	0
**Grand total**				**800.04**	**31.79**	**1,020.80**	**5.73**	**14.50**	**1,024.15**
NRV (19)				400	22	1,000	2.4	14	800
Difference between nutrient content and NRV (% of NRV)				**+400.04 (200)**	**+9.79 (129)**	**+20.80 (102)**	**+3.33 (239)**	**+0.50 (104)**	**+224.15 (128)**

## 4 Discussion

This is the first study to test the nutritional adequacy of the EAT-Lancet diet in the context of West Africa, using a region-specific food database. Contextualizing the diet to a specific region using more locally available foods increases the usefulness of the EAT-Lancet diet for national policy creation, program implementation, and dietary guidelines development. This novel use of a regional database also paves the way for continued adaptation of global guidance.

Our approach matched the suggested amounts (in g/day) for each food group in the EAT-Lancet diet with median energy and nutrient values for the same food groups identified in the WAFCT. WAFCT model energy estimates were remarkably close to EAT-Lancet diet values (2,516 vs 2,503 kcal), with similar proportions coming from plant sources. The WAFCT model provided 87 g/day of total protein and 62 g/day following PDCAAS adjustment, both of which are above the NRV of 50 g/day (21). Digestible protein is 71% of the total protein consumed, indicating the importance of correcting for digestibility particularly when recommending plant-based diets. EAT-Lancet diet provides marginally sufficient high-quality protein in the West African context; however, given that protein needs vary by weight, health status, age, sex, and activity level, protein may be inadequate for those with higher needs.

### 4.1 Dietary animal:plant (A:P) protein ratios

The unadjusted dietary A:P protein ratio in the EAT-Lancet diet was 31:69, meaning that 69% of protein came from plant-based food sources. The current average dietary A:P protein ratio in West Africa is 17:83 (10), which may also correspond with a proportion of digestible protein even lower than 71%. To align with the EAT-Lancet dietary A:P ratio, countries in West Africa would need to increase both their production and consumption of animal protein. The currently high proportion of plant-protein underscores the importance of evaluating protein digestibility. In addition to providing more high-quality protein and essential micronutrients, livestock can provide economic and food security (24). Given the association between GDP and dietary A:P protein ratio (25), economic development may also lead to increased animal protein intake.

### 4.2 Micronutrient shortfalls

Our analysis showed the WAFCT model of the EAT-Lancet diet was inadequate in vitamin A, calcium, and zinc for the general adult population. Recommending diets without adequate amounts of these nutrients is of particular concern in West Africa given the preexisting prevalence of vitamin A deficiency (26), dietary patterns high in phytates that inhibit zinc absorption (13), and limited consumption of high calcium foods like dairy (27).

These findings have consistencies with Beal et al. and raise similar concerns (13); however, there were notable methodological differences between our approaches. For example, Beal et al. (13) included foods in their cooked form, a more nutritionally realistic approach, while our analysis included foods in the form specified by the Commission or in both raw and cooked form, if unspecified. These methodological differences and different data sources between models led to different results in terms of iron adequacy. The WAFCT includes 16 iron-rich (15 mg/100 g) pearl millet items from Burkina Faso that drive up the median value used in this present analysis despite the marginal contribution of these foods to daily iron intake in a limited geography (28). The high median of iron shown for grains may overestimate dietary consumption in that the iron density of maize and rice is less than 4 mg/100 g. Despite different approaches, both analyses demonstrated folate adequacy, due to high intake of pulses.

Consistent with the observations of Beal et al. (13) we showed that calcium, zinc and vitamin A inadequacies could be remedied by the addition of small amounts of nutrient-rich foods. Both analyses corrected the EAT-Lancet diet's nutrient inadequacies by adding liver and small dried fish and replacing some whole grains with dry beans, lentils and peas. The changes applied in our West Africa-specific revision were within the EAT-Lancet diet recommended ranges, minimized animal source foods, and achieved micronutrient adequacy at 2,516 kcal/day.

The present analyses showed that the EAT-Lancet diet at 2,516 kcal/day was adequate in iron; however, the actual energy intake per day of most women and children is most likely less. If the energy intake of the EAT-Lancet diet were to be scaled down to 1,800 kcal/day, it would no longer be adequate in iron using WAFCT data. This raises specific concerns for women of reproductive age given that even adding liver and small-dried fish failed to produce a diet that was adequate in iron at 1,800 kcal/day.

The nutrient density of the diet would need to be even greater to avoid inadequacy at lower intake levels. Therefore, the Commission should consider creating various food plans that are nutritionally adequate at different levels of energy intake. Additionally, the inclusion of nutrient rich products, including fortified foods, may be important improvements for any future versions of the planetary health diet (29).

### 4.3 Micronutrient bioavailability: an unresolved issue

Another key consideration is that priority micronutrients are not equally bioavailable and have different kinetics of absorption depending on iron status of the source food and presence of anti-nutrients in the diet (22). While these factors are sometimes addressed in NRVs and other nutrition standards, they are not always considered. Importantly, iron bioavailability differs between animal-source heme iron and plant-derived non-heme iron (30). In our original WAFCT model, over 95% of the iron was non-heme. This is significant because the bioavailability of non-heme iron is highly variable and can be enhanced when co-consumed with vitamin C and animal flesh or inhibited when consumed with phytates, polyphenols, and tannins (30). Absorption rates may also be physiologically adaptable, and those who eat a highly plant-based diet may absorb more non-heme iron compared to omnivores (31). Although the NRV does adjust for iron requirements in high cereal diets based on an absorption factor of 10%, this does not provide sufficient insight into the wide variation in bioavailability by source food.

The bioavailability of calcium and zinc is also variable (32). In the modeled diet, most of the zinc came from whole grains which also contain phytates that inhibit absorption (32). Additionally, over a third of the dietary calcium came from dark green leafy vegetables, soy foods, and beans, which have a lower fractional rate of absorbable calcium than milk (33). Alternative sources of calcium in West Africa may include dried leaves of bush-okra and moringa, small fish with bones, and eggshell (34). A new algorithm was developed to estimate the bioavailability of calcium (35); however, data limitations are a current challenge to applying this algorithm to develop a calcium NRV for consumers of plant-based diets. Having standard bioavailability adjustments for minerals with variable absorption factors, similar to those for protein (i.e., PDCAAS), would be valuable for improving dietary modeling and assessment (18, 30).

### 4.4 Strengths and limitations

This study has several strengths. This EAT-Lancet diet model was based on nutrient profiles of foods listed in the FAO regional WAFCT database. As noted previously, the WAFTC database lists regional foods that are commonly consumed in a number of countries including Benin, Burkina Faso, The Gambia, Ghana, Guinea, Mali, Niger, Nigeria and Senegal. Using a database of foods that are specific to the region and are locally available is critical to evaluating dietary recommendations that have a global reach. Nutrient of WAFTC foods was measured based on their typical cultivation and preparation methods. What is more, the foods and mixed dishes were specific to the regions, commonly eaten, and by definition culturally appropriate. The WAFCT listed close to 600 foods with multiple items in each category, which increased its validity. Our novel application of the PDCAAS adjustments at the food and food group level provides insight into the EAT-Lancet diet's protein quality. These methods solidify the path for future research to perform similar analyses leveraging similar databases from other regions that may be underrepresented in the formation of global recommendations.

While extensive, the WAFCT was merely a proxy of the West Africa food supply. Comprised of 16 countries, the West African region contains various agroecological zones, all of which differ in terms of food availability, food culture, and seasonality. Rainfed agricultural systems see significant variation in the availability of certain plant-source food across the seasons. The diversity of foodways was not captured in the analysis, and food acceptance may not be equal across all food categories. Small dried fish may be more culturally imbedded than liver. Additionally, the original WAFCT has several missing data that were updated with USDA values or excluded, and there were 205 values that contained lower quality data for spread across the six micronutrients. This limits the accuracy of the data specific to West Africa given that nutrient composition may vary by region and food environment, and the direction of this bias is unknown. The present calculations were adjusted for protein digestibility but not for iron, zinc, or calcium bioavailability at the food item level. One limitation of the WAFCT is that foods are included in both raw and cooked forms. Cooking can substantially alter nutrient availability, and we used the median of the category. Due to data availability, we adjusted protein using PDCAAS, rather than the FAO-recommended DIAAS (12). We did not calculate or adjust for the likely phytate content of the plant based EAT-Lancet diet, although it has been estimated to be >2,400 mg (13). Finally, these results only report the nutritional composition of the foods included in the EAT-Lancet diet. This necessitated the exclusion of all refined, processed, and fortified foods in the WAFCT, despite their availability in the West African food supply. Similarly, the category of small ruminant animals contains only lamb in the database, when adult sheep and goats may in fact be more commonly consumed. These results must be interpreted considering the extent to which the EAT-Lancet diet currently aligns with dietary consumption. Both FAO Food Balance Sheets and survey data report adherence to the EAT-Lancet diet recommendations may be low in this region (19, 20).

## 5 Conclusions and public health impact

Based on the present analyses of the WAFCT, the EAT-Lancet diet at 2,500 kcal/day may provide sufficient protein, folate, iron, and vitamin B12 but is still inadequate in calcium, zinc, and vitamin A. At lower energy intakes, there are concerns the EAT-Lancet diet may also be inadequate in iron. These findings reinforce the importance of leveraging regional food databases to devise food plans that are both nutritionally adequate and culturally appropriate. The regional FAO database for West Africa, featuring foods commonly eaten in a number of West African Countries was especially valuable in this regard. Future iterations of the EAT-Lancet diet should account for the diversity of global food supply and nutritional needs of populations at risk.

## Data Availability

The original contributions presented in the study are included in the article/Supplementary material, further inquiries can be directed to the corresponding author.

## References

[1] Willett W Rockström J Loken B Springmann M Lang T Vermeulen S . Food in the anthropocene: the EAT–lancet commission on healthy diets from sustainable food systems. Lancet. (2019) 393:447–92. doi: 10.1016/S0140-6736(18)31788-4 30660336

[2] Lassen AD Christensen LM Trolle E. Development of a Danish adapted healthy plant-based diet based on the EAT-Lancet reference diet. Nutrients. (2020) 12:738. doi: 10.3390/nu12030738 32168838 PMC7146415

[3] Tucci M Martini D Del Bo' C Marino M Battezzati A Bertoli S . An Italian-Mediterranean dietary pattern developed based on the EAT-Lancet reference diet (EAT-IT): A nutritional evaluation. Foods. (2021) 10:558. doi: 10.3390/foods10030558 33800396 PMC8002105

[4] Bosu WK. An overview of the nutrition transition in West Africa: implications for non-communicable diseases. Proc Nutr Soc. (2015) 74:466–77. doi: 10.1017/S0029665114001669 25529539

[5] Chadare FJ Affonfere M Aidé ES Fassinou FK Salako KV Pereko K . Current state of nutrition in West Africa and projections to 2030. Glob Food Secur. (2022) 32:100602. doi: 10.1016/j.gfs.2021.100602

[6] United Nations Department of Economic and Social Affairs. Goal 2 End hunger, achieve food security and improved nutrition and promote sustainable agriculture. United Nations Sustainable Development Goals. (2024). Available online at: https://sdgs.un.org/goals/goal2#targets_and_indicators (Accessed December 23, 2025).

[7] Beal T Massiot E Arsenault JE Smith MR Hijmans RJ. Global trends in dietary micronutrient supplies and estimated prevalence of inadequate intakes. PLoS ONE. (2017) 12:e0175554. doi: 10.1371/journal.pone.0175554 28399168 PMC5388500

[8] Stevens GA Beal T Mbuya MNN Luo H Neufeld LM Addo OY . Micronutrient deficiencies among preschool-aged children and women of reproductive age worldwide: a pooled analysis of individual-level data from population-representative surveys. Lancet Glob Health. (2022) 10:e1590–9. doi: 10.1016/S2214-109X(22)00367-9 36240826 PMC10918648

[9] Drewnowski A Hooker K. The protein transition: what determines the animal-to-plant (A:P) protein ratios in global diets. Front Nutr. (2025) 12:1518793. doi: 10.3389/fnut.2025.1518793 40013163 PMC11860088

[10] Food and Agriculture Organization of the United Nations. Daily Protein Supply from Animal and Plant-Based Foods. Processed by Our World in Data (2023). Available online at: https://ourworldindata.org/grapher/daily-protein-supply-from-animal-and-plant-based-foods (Accessed December 23, 2025).

[11] Schaafsma G. The protein digestibility–corrected amino acid score. J Nutr. (2000) 130:1865S−1867S. doi: 10.1093/jn/130.7.1865S 10867064

[12] FAO Expert Consultation. Dietary protein quality evaluation in human nutrition. Food Agric Organ U N. (2013) 92:1–66. Available online at: https://www.fao.org/4/i3124e/i3124e.pdf (Accessed December 23, 2025). 26369006

[13] Beal T Ortenzi F Fanzo J. Estimated micronutrient shortfalls of the EAT–Lancet planetary health diet. Lancet Planet Health. (2023) 7:e233–7. doi: 10.1016/S2542-5196(23)00006-2 36889864

[14] Vincent A. FAO/INFOODS Food Composition Table for Western Africa (2019)/Table de composition des aliments FAO/INFOODS pour l'Afrique de l'Ouest (2019): User Guide & Condensed Food Composition Table/Guide d'utilisation & table de composition des aliments condensée. Rome, Italy: FAO (2020). 556 p. Available online at: https://www.fao.org/documents/card/en?details=ca7779b (Accessed December 23, 2025).

[15] U.S. Department of Agriculture. USDA Food and Nutrient Database for Dietary Studies 2017- 348 2018. Baltimore, MD: U.S. Department of Agriculture (2018). p. 1–39. https://agdatacommons.nal.usda.gov/articles/dataset/Food_and_Nutrient_Database_for_Dietary_Studies_FNDDS_/24660933?file=43365429 (Accessed December 23, 2025).

[16] Lara-Arevalo J Laar A Chaparro MP Drewnowski A. Nutrient-dense African Indigenous vegetables and grains in the FAO food composition table for Western Africa (WAFCT) identified using nutrient-rich food (NRF) scores. Nutrients. (2024) 16:2985. doi: 10.3390/nu16172985 39275300 PMC11397376

[17] FAO. Minimum Dietary Diversity for Women. Rome: FAO (2021). Available online at: https://openknowledge.fao.org/items/dee8f29f-cf6c-4dcb-9cb9-05c263e7219b (Accessed December 23, 2025).

[18] Beal T Ortenzi F. Priority micronutrient density in foods. Front Nutr. (2022) 9:806566. doi: 10.3389/fnut.2022.806566 35321287 PMC8936507

[19] Ali Z Scheelbeek PFD Felix J Jallow B Palazzo A Segnon AC . Adherence to EAT-Lancet dietary recommendations for health and sustainability in the Gambia. Environ Res Lett. (2022) 17:104043. doi: 10.1088/1748-9326/ac9326 36238079 PMC9536464

[20] Food and Agriculture Organization of the United Nations EAT-Lancet Commission. How do Actual Diets Compare to the EAT-Lancet Diet?. Processed by Our World in Data. (2021). Available online at: https://ourworldindata.org/grapher/eat-lancet-diet-comparison (Accessed December 23, 2025).

[21] Lewis J. Codex Nutrient Reference Values. Rome: FAO and WHO (2019). Available online at: https://openknowledge.fao.org/server/api/core/bitstreams/2033128c-4d26-47d0-8f67-2f736c4a1d29/content (Accessed December 23, 2025).

[22] Leonard UM Leydon CL Arranz E Kiely ME. Impact of consuming an environmentally protective diet on micronutrients: a systematic literature review. Am J Clin Nutr. (2024) 119:927–48. doi: 10.1016/j.ajcnut.2024.01.014 38569787

[23] Fulgoni VL Keast DR Drewnowski A. Development and validation of the nutrient-rich foods index: a tool to measure nutritional quality of foods. J Nutr. (2009) 139:1549–54. doi: 10.3945/jn.108.101360 19549759

[24] FAO. Contribution of Terrestrial Animal Source Food to Healthy Diets for Improved Nutrition and Health Outcomes—An Evidence and Policy Overview on the State of Knowledge and Gaps. Rome: Food and Agriculture Organization of the United Nations (2023). doi: 10.4060/cc3912en

[25] Drewnowski A. Alternative proteins in low- and middle-income countries (LMIC) face a questionable future: will technology negate Bennett's law? Curr Dev Nutr. (2023) 8:101994. doi: 10.1016/j.cdnut.2023.101994 38476727 PMC10926128

[26] Wirth JP Petry N Tanumihardjo SA Rogers LM McLean E Greig A . Vitamin A supplementation programs and country-level evidence of vitamin A deficiency. Nutrients. (2017) 9:190. doi: 10.3390/nu9030190 28245571 PMC5372853

[27] Food and Agriculture Organization of the United Nations. Dietary Compositions by Commodity Group. Processed by Our World in Data (2024). Available online at: https://ourworldindata.org/grapher/dietary-compositions-by-commodity-group?country=Western+Africa+%28FAO%29ÕWID_NAMÕWID_WRL (Accessed December 23, 2025).

[28] Hama-Ba F Mouquet-Rivier C Diawara B Weltzien E Icard-Vernière C. Traditional African dishes prepared from local biofortified varieties of pearl millet: acceptability and potential contribution to iron and zinc intakes of Burkinabe young children. Front Nutr. (2019) 6:115. doi: 10.3389/fnut.2019.00115 31475149 PMC6702452

[29] EAT-Lancet 2.0 Commissioners. EAT–*lancet* commission 2.0: securing a just transition to healthy, environmentally sustainable diets for all. Lancet. (2023) 402:352–4. doi: 10.1016/S0140-6736(23)01290-4 37442146

[30] van Wonderen D Melse-Boonstra A Gerdessen JC. Iron bioavailability should be considered when modeling omnivorous, vegetarian, and vegan diets. J Nutr. (2023) 153:2125–32. doi: 10.1016/j.tjnut.2023.05.011 37182693

[31] López-Moreno M Viña I Marrero-Fernández P Galiana C Bertotti G Roldán-Ruiz A . Dietary adaptation of non-heme iron absorption in vegans: a controlled trial. Mol Nutr Food Res. (2025) 69:e70096. doi: 10.1002/mnfr.70096 40320969 PMC12189169

[32] Gibson RS Raboy V King JC. Implications of phytate in plant-based foods for iron and zinc bioavailability, setting dietary requirements, and formulating programs and policies. Nutr Rev. (2018) 76:793–804. doi: 10.1093/nutrit/nuy028 30010865

[33] Shkembi B Huppertz T. Calcium absorption from food products: food matrix effects. Nutrients. (2021) 14:180. doi: 10.3390/nu14010180 35011055 PMC8746734

[34] Bartter J Diffey H Yeung YH O'Leary F Häsler B Maulaga W . Use of chicken eggshell to improve dietary calcium intake in rural sub-Saharan Africa. Matern Child Nutr. (2018) 14:e12649. doi: 10.1111/mcn.12649 30332539 PMC6221107

[35] Weaver CM Wastney M Fletcher A Lividini K. An algorithm to assess calcium bioavailability from foods. J Nutr. (2024) 154:921–7. doi: 10.1016/j.tjnut.2023.12.005 38072154

