# Supplementary material for: Adapting the EAT-Lancet diet for West Africa: protein quality and micronutrient inadequacies improved through nutrient dense foods
Source: Front Nutr. 2026 Jan 14;12:1673484. doi: 10.3389/fnut.2025.1673484 (PMC12846968; doi:10.3389/fnut.2025.1673484)
Supplement: Supplementary file 1 [file Table_1.docx]

**Supplemental Tables**

**Supplemental Table 1.** Specific PDCAAS values for foods and food groups

| **Food group** | **Foods** | **PDCAAS Values** | **Source** |
| --- | --- | --- | --- |
| Legumes and pulses | Black beans | 0·61 | A |
|  | Brown beans | 0·61 | A |
|  | Kidney beans | 0·61 | A |
|  | White beans | 0·61 | A |
|  | Navy beans | 0·61 | A |
|  | Pinto beans | 0·61 | A |
|  | Red beans | 0·61 | A |
|  | Cowpeas | 0·38 | A |
|  | Lentils | 0·52 | A |
|  | Soybeans | 0·91 | B |
|  | Peas | 0·69 | A |
|  | Cooked peas | 0·6 | C |
|  | Pigeon pea | 0·78 | A |
|  | Bambara bean | 0·78 | A |
|  | Legumes and peas | 0·7 | A |
|  | Legumes | 0·74 | D |
| Nuts and seeds | Nuts and seeds | 0·47 (mean) | D |
| Grains | Corn | 0·47 | E |
|  | Corn flour | 0·44 | D |
|  | Cornmeal | 0·37 | F |
|  | Pearl Millet | 0·2 | F |
|  | Oats | 0·66 | F |
|  | Breakfast cereal | 0·8 | G |
|  | Rice | 0·81 | H |
|  | Cooked Rice | 0·62 | C |
|  | Rice, brown | 0·61 | D |
|  | Sorghum flour | 0·46 | D |
|  | Wheat | 0·46 | E |
|  | Wheat bread | 0·37 | F |
|  | Wheat bran | 0·67 | D |
|  | Wheat flour | 0·47 | D |
|  | Other cereals | 0·69 | D |
| Milk, dairy and eggs | Milk | 1 | I |
|  | Milk Cream | 0·8 | F |
|  | Cheese | 0·99 | J |
| Egg | Egg | 1 | D |
| Meat and poultry | Beef/ tripes/ giblets | 0·94 | D |
|  | Chicken | 0·94 | D |
|  | Pork | 0·98 | F |
|  | Sausage | 0·94 | D |
|  | Other meats (Ostrich, crocodile, camel) | 0·94 | D |
|  | Insects (cricket) | 0·76 | K |
|  | Termites | 0·9 | K |
|  | Mixed dishes with beef | 0·83 (mean) | J |
| Fish and seafood | Sardine | 1 | F |
|  | Shrimp/ crab/ sea snail/ tilapia/ clams | 0·94 | D |
|  | Mackerel | 0·94 | F |
|  | Tilapia | 0·94 | D |
|  | Tuna | 1 | F |
|  | Other fish | 0·94 | D |
| Vegetables | Cabbage | 0·62 | D |
|  | Amaranth | 0·73 | D |
|  | Potato | 0·81 | F |
|  | Eggplant | 0·55 | D |
|  | Spinach | 0·75 | D |
|  | Lettuce | 0·16 | D |
|  | Cucumber | 0·3 | D |
|  | Tomato | 0·39 | D |
|  | Pumpkin | 0·29 | D |
|  | Onion | 0·39 | D |
|  | Carrot | 0·74 | D |
|  | Other tubers | 0·74 | D |
|  | Other leaves and vegetables | 0·73 | D |
|  | Orange | 0·42 | D |
| Fruits | Watermelon | 0·46 | D |
|  | Banana | 0·75 | D |
|  | Apple | 0·72 | D |
|  | Grape | 0·27 | D |
|  | Other fruit | 0·64 | D |
|  | Date | 0·31 | D |
|  | Avocado | 0·71 | D |
|  | Dried fruits | 0·48 | D |

**Sources**

1. Semba, R. D., Ramsing, R., Rahman, N., Kraemer, K., & Bloem, M. W. (2021). Legumes as a sustainable source of protein in human diets. *Global Food Security*, *28*, 100520. <https://doi.org/10.1016/j.gfs.2021.100520>
2. Schaafsma, G. (2000). The Protein Digestibility–Corrected Amino Acid Score. The Journal of Nutrition, 130(7), 1865S-1867S. <https://doi.org/10.1093/jn/130.7.1865S>
3. van Vliet, S., Burd, N. A., & van Loon, L. J. C. (2015). The Skeletal Muscle Anabolic Response to Plant- versus Animal-Based Protein Consumption. *The Journal of Nutrition*, *145*(9), 1981–1991. <https://doi.org/10.3945/jn.114.204305>
4. Suárez López, M. M., Kizlansky, A., & López, L. B. (2006). Evaluación de la calidad de las proteínas en los alimentos calculando el escore de aminoácidos corregido por digestibilidad [Assessment of protein quality in foods by calculating the amino acids score corrected by digestibility]. *Nutricion Hospitalaria*, *21*(1), 47–51.
5. Ertl, P., Knaus, W., & Zollitsch, W. (2016). An approach to including protein quality when assessing the net contribution of livestock to human food supply. *Animal: An International Journal of Animal Bioscience*, *10*(11), 1883–1889. <https://doi.org/10.1017/S1751731116000902>
6. Boye, J., Wijesinha-Bettoni, R., & Burlingame, B. (2012). Protein quality evaluation twenty years after the introduction of the protein digestibility corrected amino acid score method. *The British Journal of Nutrition*, *108 Suppl 2*, S183-211. <https://doi.org/10.1017/S0007114512002309>
7. Rutherfurd, S. M., Fanning, A. C., Miller, B. J., & Moughan, P. J. (2015). Protein digestibility-corrected amino acid scores and digestible indispensable amino acid scores differentially describe protein quality in growing male rats. *The Journal of Nutrition*, *145*(2), 372–379. <https://doi.org/10.3945/jn.114.195438>
8. Nitrayová, S., Brestenský, M., & Patráš, P. (2018). Comparison of two methods of protein quality evaluation in rice, rye and barley as food protein sources in human nutrition. *Slovak Journal of Food Sciences*, *12*(1), 762–766. <https://doi.org/10.5219/991>
9. Hoffman, J. R., & Falvo, M. J. (2004). Protein – Which is Best? Journal of Sports Science & Medicine, 3(3), 118–130.
10. Food and Drug Administration. (1993). True Protein Digestibility Value of Common Foods. Federal Register, 58(3), 2193–2195.
11. Noyens, I., Van Miert, S., Brombach, C., Rossi, M., Haas, N., Beckman, M., Alvarez, C., Neves, E., Naranjo-Guevara, N., Garrelts, K., Floto-Stammen, S., & Roosen, M. (2021). Digestibility of insect proteins and insect-based products: Literature review. Lille : ValuSect. https://library.wur.nl/WebQuery/titel/2330903

**Supplemental Table 2.** Energy, protein and micronutrient intake of the EAT-Lancet diet when matching energy intake by food group, based on WAFCT medians

|  |  |  |  | **Model-WAFCT Totals** | | | | | | | | |
| --- | --- | --- | --- | --- | --- | --- | --- | --- | --- | --- | --- | --- |
| **EAT-Lancet Food Group** | **EAT-Lancet Food Subgroup** | **Mass intake of WAFCT adjusted for EAT-Lancet kcal (g/d)** | **EAT-Lancet kcal/day** | **Energy (kcal/d)** | **Protein (g/d)** | **PDCAAS Protein (g/d)** | **Folate, DFE (µg/d)** | **Iron (mg/day)** | **Calcium (mg/d)** | **Vitamin B12 (µg/d)** | **Zinc (µg/d)** | **Vitamin A, RAE (µg/d)** |
| **Whole Grains** | Whole grains (rice, wheat, corn and other) | 232 | 811 | 811 | 21·78 | 9·52 | 164·52 | 15·76 | 53·29 | 0 | 5·10 | 0·00 |
| **Tubers or Starchy Vegetables** | Tubers or starchy vegetables (potatoes and cassava) | 30 | 39 | 39 | 0·52 | 0·38 | 5·29 | 0·29 | 4·53 | 0 | 0·13 | 1·51 |
| **Vegetables** | Dark green leafy vegetables | 50 | 23 | 23 | 2·13 | 1·55 | 33·00 | 1·85 | 124·50 | 0 | 0·34 | 105·00 |
|  | Other vegetables | 64 | 25 | 25 | 0·97 | 0·56 | 13·14 | 0·64 | 18·59 | 0 | 0·17 | 5·77 |
|  | Red and orange vegetables | 85 | 30 | 30 | 0·80 | 0·59 | 12·68 | 1·52 | 27·89 | 0 | 0·25 | 107·32 |
| **Fruits** | Fruits | 179 | 126 | 126 | 1·70 | 1·01 | 24·13 | 1·16 | 38·43 | 0 | 0·27 | 6·26 |
| **Dairy Foods** | Dairy foods (whole milk or equivalents) | 239 | 153 | 153 | 7·89 | 7·89 | 19·13 | 0·24 | 284·48 | 1·41 | 1·51 | 100·41 |
| **Protein Sources** | Beef and lamb | 8 | 15 | 15 | 2·11 | 1·98 | 0·69 | 0·26 | 1·47 | 0·20 | 0·29 | 0·69 |
|  | Pork | 4 | 15 | 15 | 0·90 | 0·88 | 0·34 | 0·09 | 0·59 | 0·02 | 0·09 | 0·08 |
|  | Chicken and other poultry | 41 | 62 | 62 | 10·33 | 9·71 | 2·03 | 0·57 | 5·27 | 0·15 | 0·82 | 8·92 |
|  | Eggs | 11 | 19 | 19 | 1·35 | 1·35 | 6·88 | 0·25 | 7·46 | 0·18 | 0·17 | 12·59 |
|  | Fish | 34 | 40 | 40 | 7·86 | 7·39 | 4·05 | 0·51 | 20·25 | 1·15 | 0·37 | 4·73 |
|  | Dry beans, lentils, and peas | 54 | 172 | 172 | 11·15 | 7·69 | 176·82 | 2·46 | 39·65 | 0 | 1·29 | 0·54 |
|  | Soy foods | 29 | 112 | 112 | 10·26 | 9·34 | 111·27 | 2·02 | 60·61 | 0 | 1·40 | 0 |
|  | Peanuts | 25 | 142 | 142 | 5·54 | 2·60 | 27·19 | 0·89 | 12·48 | 0 | 0·65 | 0·49 |
|  | Tree nuts | 27 | 149 | 149 | 3·86 | 1·82 | 15·78 | 1·50 | 15·92 | 0 | 0·89 | 0 |
| **Added Fats** | Palm oil | 7 | 60 | 60 | 0 | 0 | 0 | 0·01 | 0·07 | 0 | 0 | 0 |
|  | Unsaturated oils | 39 | 354 | 354 | 0 | 0 | 0 | 0·03 | 0 | 0 | 0 | 0 |
|  | Dairy fats (included in milk) | 0 | 0 | 0 | 0 | 0 | 0 | 0 | 0 | 0 | 0 | 0 |
|  | Lard or tallow | 0 | 36 | 0 | 0 | 0 | 0 | 0 | 0 | 0 | 0 | 0 |
| **Added Sugar** | All sugars | 37 | 120 | 120 | 0·06 | 0·15 | 0·74 | 0·22 | 4·05 | 0 | 0·07 | 0 |
| **Plant Source Total** |  | 857 | 2163 | 2163 | 58·77 | 35·21 | ·· | ·· | ·· | ·· | ·· | ·· |
| **Animal Source Total** |  | 336 | 340 | 304 | 30·45 | 29·21 | ·· | ·· | ·· | ·· | ·· | ·· |
| **Grand total after matching energy** |  |  | 2503 | 2467 | 89·22 | 64·43 | 617·66 | 30·27 | 719·54 | 3·10 | 13·82 | 354·31 |

**Supplemental Table 3.** FAO Codex Alimentarius nutrient reference values (NRV) for protein and priority micronutrients^1^

| **Nutrient** | **Daily NRV** |
| --- | --- |
| Protein | 50 g |
| Calcium | 1000 mg |
| Folate (DFE) | 400 μg |
| Iron | 22 mg |
| Vitamin A (RAE) | 800 μg |
| Vitamin B12 | 2·4 μg |
| Zinc | 14 μg |

^1^ Lewis J. Codex nutrient reference values [Internet]. Rome: FAO and WHO; 2019. Available from: https://openknowledge.fao.org/server/api/core/bitstreams/2033128c-4d26-47d0-8f67-2f736c4a1d29/content

**Supplemental Table 4.** Nutrient density of the EAT-Lancet diet and subgroups based on WAFCT median values·

| EAT-Lancet  food group | EAT-Lancet food subgroup | NR6 (SD) | LIM (SD) | NRF6·3 (SD) |
| --- | --- | --- | --- | --- |
| Whole Grains | Whole grains (rice, wheat, corn and other) | 29·23 (12·15) | 1·35 (1·32) | 27·88 (11·95) |
| Tubers or Starchy Vegetables | Tubers or starchy vegetables (potatoes and cassava) | 21·73 (20·68) | 2·71 (4·81) | 19·02 (21·94) |
| Vegetables | Dark green leafy vegetables | 207·26 (84·53) | 3·93 (2·94) | 203·33 (83·44) |
|  | Other vegetables | 61·12 (33·51) | 2·34 (1·70) | 58·78 (33·23) |
|  | Red and orange vegetables | 80·72 (31·98) | 5·53 (12·91) | 75·19 (32·83) |
| Fruits | Fruits | 24·72 (16·20) | 2·09 (7·07) | 22·63 (18·72) |
| Dairy Foods | Dairy foods (whole milk) | 88·46 (0) | 19·54 (0) | 68·92 (0) |
| Protein Sources | Beef and lamb | 112·52 (47·16) | 16·81 (11·73) | 95·71 (51·11) |
|  | Beef and lamb liver | 315·25 (25·92) | 8·03 (0·90) | 307·22 (26·04) |
|  | Pork | 28·67 (7·96) | 15·43 (3·07) | 13·24 (10·81) |
|  | Chicken and other poultry | 106·38 (104·04) | 9·35 (8·63) | 97·03 (104·96) |
|  | Chicken liver | 398·84 (3·73) | 7·60 (0·50) | 391·24 (3·30) |
|  | Eggs | 109·03 (33·45) | 13·86 (1·48) | 95·16 (33·45) |
|  | Fish | 149·65 (55·37) | 8·86 (9·46) | 140·78 (54·54) |
|  | Small dried fish | 207·52 (0) | 6·72 (0) | 200·80 (0) |
|  | Dry beans, lentils, and peas | 50·18 (15·32) | 1·54 (0·83) | 48·65 (15·96) |
|  | Soy foods | 67·54 (2·60) | 3·15 (0·31) | 64·39 (2·61) |
|  | Peanuts | 19·19 (7·46) | 7·55 (1·53) | 11·64 (8·96) |
|  | Tree nuts | 21·84 (8·57) | 13·83 (14·97) | 8·01 (22·00) |
| Added Fats | Palm oil | 0·10 (0·06) | 37·21 (11·86) | -37·11 (11·91) |
|  | Unsaturated oils | 0·12 (0·16) | 11·35 (6·37) | -11·23 (6·24) |
|  | Dairy fats (included in milk) | 14·52 (1·66) | 37·71 (2·94) | -23·19 (2·34) |
|  | Lard or tallow | ·· | ·· | ·· |
| Added Sugar | All sugars | 2·03 (1·76) | 37·12 (22·00) | -35·08 (22·17) |

**Supplemental Table 5.** Micronutrient density of additional subcategories of revised EAT-Lancet diet and their respective parent categories

| **EAT-Lancet Food Subcategory** | **Folate (µg)** | **Iron (mg)** | **Calcium (mg)** | **Vitamin B12 (µg)** | **Zinc (µg)** | **Vitamin A (RAE) (µg)** |
| --- | --- | --- | --- | --- | --- | --- |
| Chicken and other poultry | 4·00 | 1·20 | 13·00 | 0·31 | 1·77 | 14·00 |
| Chicken liver | 1000·00 | 11·85 | 7·00 | 22·50 | 3·69 | 8110·00 |
| Beef | 11·00 | 1·80 | 20·00 | 1·80 | 4·76 | 10·0 |
| Beef and lamb liver | 235·00 | 12·50 | 21·50 | 100·00 | 4·14 | 18300·00 |
| Fish | 8·50 | 1·50 | 60·00 | 2·95 | 1·11 | 14·00 |
| Small dried fish | 46·00 | 7·40 | 1939·00 | 5·00 | 7·67 | 141·00 |
